# AI solutions to the radiology workforce shortage

**DOI:** 10.1038/s44401-025-00023-6

**Published:** 2025-05-27

**Authors:** Andrew B. Jing, Naveen Garg, Jiajie Zhang, Jeffrey J. Brown

**Affiliations:** 1 Department of Abdominal Imaging, Division of Diagnostic Imaging, University of Texas MD Anderson Cancer Center, Houston, TX USA; 2 Bradley McWilliams School of Biomedical Informatics, University of Texas Health Science Center at Houston, Houston, TX USA

**Keywords:** Information systems and information technology, Health occupations, Health care

## Abstract

The growing demand for imaging services in the U.S., driven by an aging population and the rise in chronic diseases, has contributed to a significant radiology workforce shortage. Simultaneously, supply-side constraints, including limited radiology residency positions and substantial retirements, have exacerbated this gap. These imbalances increase patient wait times, risk diagnostic delays, and contribute to radiologist burnout. Artificial intelligence (AI) offers potential solutions by addressing three primary areas: demand management, workflow efficiency, and capacity building. First, to manage demand, AI tools can leverage predictive analytics and decision-support systems to reduce unnecessary imaging and prioritize high-value imaging examinations. Next, AI can streamline tasks and boost efficiency with applications such as automated scheduling, assisted report generation, and image quality checks. Finally, by enhancing education, facilitating remote collaboration, improving patient communication, and offering advanced image interpretation assistance, AI can expand radiologists’ capabilities, improve retention, and enhance long-term workforce sustainability. By integrating these approaches, radiology can address workforce shortages while upholding the highest standards of patient care.

## Introduction to the radiology workforce shortage

The field of radiology is experiencing unprecedented demand, driven by an aging population, the increasing prevalence of chronic diseases, and advances in imaging technologies. However, this surge in demand has not been matched by a corresponding increase in the number of radiologists, leading to a significant workforce shortage^1,2^. The implications of this shortage are far-reaching. Patients may face longer wait times for their imaging examinations, which can delay diagnosis and treatment, while radiologists face mounting pressure to maintain accuracy despite increasing workloads. Artificial intelligence (AI) has emerged as a promising tool to address these challenges by enhancing efficiency and productivity^3^. This paper explores the radiology workforce shortage in the U.S. and the potential of AI to mitigate this problem.

## Demand-side factors

**Fig. 1:**
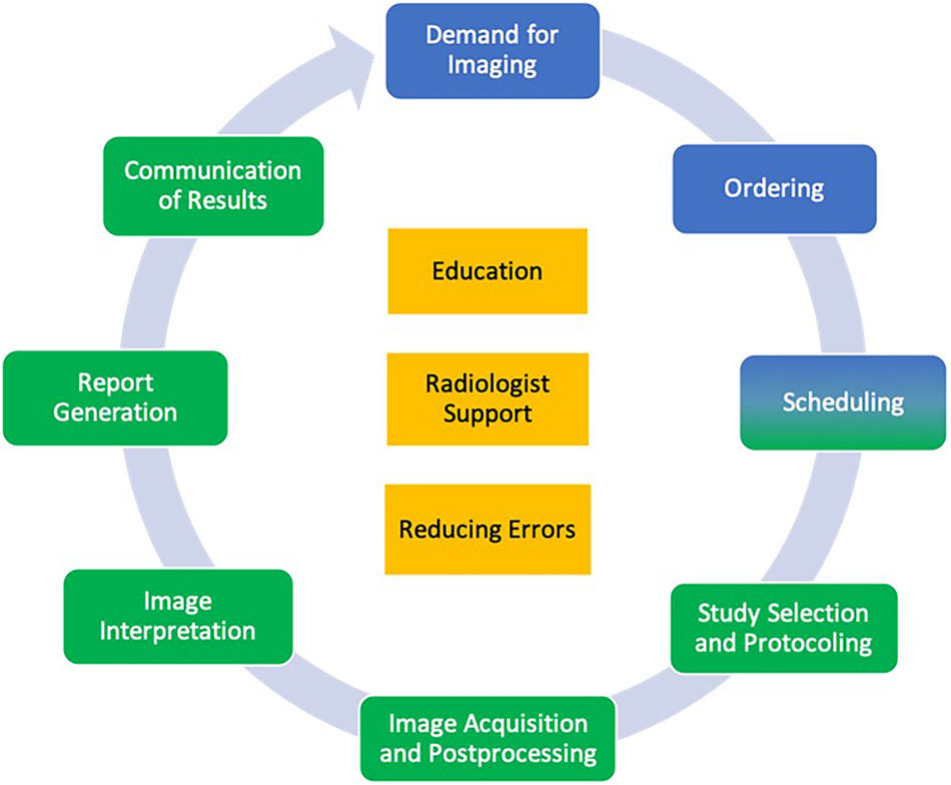
Radiology workflow cycle: AI opportunities to enhance efficiency and support workforce resilience. Blue areas indicate stages where AI can assist with demand management. Green areas highlight opportunities for efficiency gains, while yellow areas represent points where AI can strengthen workforce capacity and enhance radiologist performance.

The radiology workflow cycle starts with demand for medical imaging (Fig. 1: demand for imaging). A major factor contributing to the surge in demand for imaging studies is the growth and aging of the U.S. population^4^. According to the U.S. Census Bureau, the population grew by 7.4% from 2010 to 2020, while the percentage of individuals aged 65 or older increased by 38.6%^5^. The aging population, along with risk factors such as smoking and obesity, contributes to the rising prevalence of chronic diseases, including heart disease, cancer, and dementia.

The projected aging of the U.S. population over the coming decades is expected to drive additional growth in demand for medical imaging to help meet the healthcare needs of an older demographic^6^. While some diseases may decrease in prevalence due to medical breakthroughs and advances in population health, most chronic conditions are expected to continue their upward trends. For example, the number of new cancer cases per year in the U.S. is predicted to grow from 2.4 million cases in 2022 to 3.4 million cases in 2045, a 42% increase^7^.

## Supply-side factors

While demand for medical imaging has increased, the number of radiologists has not kept pace. There are about 34,000 radiologists practicing in the U.S.^8^. Among these, 16% or more are working part-time, and 32% are over age 55, suggesting that future retirements could exacerbate the workforce shortage^9,10^. The American College of Radiology (ACR) job board has 1930 positions posted currently^11^; however, fewer than 1400 radiology residents match per year^12^. As a result, many private practices and academic departments are short-staffed and in a perpetual recruiting mode.

The need to train additional radiologists is clear, yet structural, financial, and time-related challenges persist. Medicare and Medicaid, the main funding sources for U.S. graduate medical education (GME), have been constrained since the Balanced Budget Act of 1997, which capped fundable residency positions at 1996 levels^13^. Any residency expansion beyond this cap requires alternative funding sources.

This physician shortage extends beyond radiology, with the AAMC predicting a shortfall of up to 121,900 physicians by 2032^14^. To help address this, the Resident Physician Shortage Reduction Act of 2023 proposes to increase the number of Medicare-supported residency slots by 2000 new positions annually over five years^15^. However, given that this increase covers all medical specialties and the path to becoming a radiologist typically includes an internship, a four-year residency, and often a fellowship, this measure appears insufficient to meet the specific needs of radiology.

In the future, physicians in other specialties could assume a more prominent role in interpreting medical images. For example, cardiologists, neurologists, pulmonologists, and vascular surgeons, who already interpret imaging examinations and perform image-guided procedures, could take a greater share of the overall imaging volume if the radiologist shortage remains unresolved. Alternatively, the role of nonphysician practitioners, such as nurse practitioners, physician assistants, and advanced sonographers, could be expanded to include image interpretation, although this may raise concerns regarding training and quality assurance.

## Consequences of the workforce shortage

The radiology workforce shortage poses a risk to patient care. Operational inefficiencies and understaffing can lead to overworked radiologists, while error rates among radiologists have been linked to excessive workload, and visual and mental fatigue^16,17^. Workforce shortages can also lead to imaging delays, which may contribute to prolonged hospital stays and delays in diagnosis and treatment^18^.

To manage rising diagnostic workloads, radiology practices often extend work hours and increase productivity expectations. While this may provide short-term relief, it can also exacerbate job dissatisfaction and burnout, factors that contribute to higher attrition and perpetuate the cycle of understaffing. Burnout rates are estimated at 46% in private practice and 37.4% in academic settings^19,20^. In response, some practices have implemented flexible scheduling, part-time and remote work options, and teleradiology services to help alleviate the burden^2,9^. However, these efforts primarily address symptoms rather than the underlying issue: a persistent shortage of radiologists. As imaging volumes continue to rise and the radiologist workforce remains limited, there is a growing imperative to adopt AI tools that can enhance efficiency and support sustainable clinical productivity.

AI promises a wide array of improvements in the field of radiology. We organize these applications into three domains: applications that help to manage demand, optimize workflow, and build capacity.

## Demand management

Ensuring that each patient receives the most appropriate imaging study at the right time is a cornerstone of high-quality clinical care. Unnecessary imaging examinations that do not influence clinical decisions may increase patient anxiety, drive up healthcare costs, and overburden radiologists without improving patient outcomes^21–23^. Estimates of overutilization vary greatly depending on the imaging modality and clinical context. A systematic review found an 11.2% median prevalence of overuse of diagnostic imaging examinations^24^. Other studies have estimated overutilization rates of 20–50%^22,25^. Addressing the challenge of imaging overutilization is essential to alleviating strain on healthcare systems. AI can help address these issues and improve efficiency by reducing unnecessary studies through evidence-based guidelines and predictive analytics^26^.

### Decision support and feedback

AI can streamline the ordering of imaging examinations by providing clinical decision support (CDS) tools that guide providers toward appropriate imaging choices based on patient data and evidence-based guidelines (Fig. 1: ordering). AI tools based on the ACR Appropriateness Criteria, for example, can be used to improve adherence to clinical guidelines. These are evidence-based recommendations to help healthcare providers make the most appropriate imaging decisions for specific clinical conditions^27^. AI-powered tools based on these criteria can guide referring physicians in ordering imaging studies judiciously, and help radiologists identify redundant, unnecessary, or low-value imaging orders^28,29^. AI can also potentially minimize the need for follow-up or repeat imaging by enhancing diagnostic confidence. Equivocal findings may necessitate additional imaging, placing greater demands on imaging resources. By providing precise quantification of disease markers, AI may help radiologists identify clinically significant changes and recommend repeat imaging only when necessary and likely to yield actionable information.

### Triaging and prioritization

AI also has the potential to optimize triaging and scheduling processes (Fig. 1: scheduling)^30,31^. Non-urgent or routine cases can be flagged for imaging at off-peak hours, reducing demand spikes that can overwhelm radiology departments^32,33^. AI algorithms can dynamically adjust schedules to prioritize urgent cases while directing non-urgent requests to alternate or lower-resource imaging modalities when appropriate. Predictive analytics tools can forecast demand peaks and help distribute workloads more evenly, thereby improving efficiency and preventing bottlenecks^34–36^.

### Chronic disease management

AI-driven predictive analytics can address overutilization at a more systemic level by targeting long-term imaging needs. Coordinated AI agents can monitor and manage chronic diseases by continuously analyzing data from multiple sources to track disease progression, predict exacerbations, and remind patients about screening examinations or medications^37^. By predicting chronic disease risks and progression, AI can help identify patients who may benefit from early interventions, such as lifestyle changes or pharmacological treatments that may reduce the need for frequent follow-up imaging^38^. For example, in patients with chronic diseases like diabetes, AI can predict disease trajectories, make pharmacotherapy recommendations, and suggest preventative measures to delay progression to more imaging-intensive stages of the disease^39,40^. This proactive approach can lead to a reduction in overall imaging volumes while maintaining high-quality care for patients.

### Resource allocation for public health

Predictive analytics also plays a role in optimizing the allocation of imaging resources across healthcare systems. By analyzing trends in seasonal or population health changes, AI can forecast demand and help systems prepare for potential demand surges. AI tools can help distribute resources more evenly and efficiently, avoiding delays and ensuring equitable access to imaging services.

### Optimizing screening programs

AI can also play a role in population-wide screening efforts. By dynamically adjusting screening frequencies based on individual risk factors, AI can target high-risk populations more effectively while reducing unnecessary screenings in low-risk groups. Successful AI tools may enable an individualized, risk-based approach to improve the overall yield of screening programs. For example, promising progress has been made in integrating AI into mammography screening, among other cancer screening applications^41–43^.

To address overutilization, radiology needs tools to improve decision support, optimize scheduling, enhance predictive analytics, and reduce unnecessary follow-up imaging. AI-powered tools can help in these efforts, supporting the appropriate deployment of imaging resources and prioritizing high-value care. While demand management focuses on controlling and streamlining imaging requests, radiology workforce challenges also require solutions on the supply side. In the following sections, we will explore how AI can enhance workflow efficiency, helping radiologists perform their work faster and more productively, and subsequently, how it can build capacity by augmenting radiologists’ abilities and expanding the overall reach of radiology services.

## Workflow efficiency

AI solutions can enhance the efficiency of radiology workflows, allowing radiologists to perform their work faster with a high degree of accuracy^44^. In a recent study, an advanced AI model was shown to deliver consistent accuracy in detecting 21 key findings on abdominal and pelvic CT scans with an average AUC of 0.923, with exceptional performance in diagnosing some acute conditions, such as small bowel obstruction (AUC 0.958) and acute pancreatitis (AUC 0.961)^45^.

AI can also improve efficiency in image acquisition and image processing. For example, deep learning techniques can shorten MRI scan times by allowing faster image acquisition, rapid image reconstruction, and more efficient use of limited data, often without sacrificing image quality (Fig. 1: image acquisition and postprocessing)^46^. These tools can help optimize processes and minimize delays, enabling radiologists to better meet growing demand within the current scope of their practice^47^.

### Dynamic scheduling

AI-assisted scheduling can optimize imaging resource use and patient flow (Fig. 1: scheduling). By analyzing historical modality usage patterns, patient data, and radiologist availability, these tools can reduce scheduling conflicts, prevent idle equipment time, and prioritize urgent cases based on clinical need^33,36^. Predictive models can also forecast patient attendance, enabling the system to send targeted reminders to reduce no-show rates^34^. These enhancements ensure that radiologists and imaging equipment are used as efficiently as possible, with measurable success reflected in shorter patient wait times and higher equipment utilization rates.

### Automation of routine tasks

AI excels at automating repetitive but essential tasks, such as image quality assurance^48^. These tools can identify motion artifacts, suboptimal image contrast, and incorrect positioning before images are sent for interpretation, ensuring that only diagnostically useful studies reach the radiologist^49^. AI-automation has the potential to reduce turnaround times and lower rates of rejected or repeated imaging studies.

### Clinical history summarization

Reviewing patient medical histories is a time-consuming but essential component of radiology practice. Large language models (LLMs) can help streamline this process by extracting pertinent clinical information from electronic health records and generating concise and accurate summaries. These summaries can highlight critical findings or longitudinal trends that help inform interpretation^50^. Success in this area can be measured by time saved in medical record review and improvements in diagnostic accuracy and efficiency. AI research is also developing the use of temporal reasoning to organize clinical events chronologically and track disease progression or treatment response over time^51,52^. For instance, in chronic disease cases, AI can summarize imaging findings over multiple visits, presenting key changes in an intuitive timeline for the radiologist^52^.

### Protocoling

AI can assist with protocoling CT and MRI exams by analyzing clinical information, such as the exam indication, patient history, laboratory, pathology, and genomic results, to recommend the most appropriate imaging protocol for each patient (Fig. 1: study selection and protocoling)^53,54^. This ensures the exam is tailored to the clinical question, improving diagnostic accuracy and efficiency while minimizing unnecessary scans, contrast use, and radiation exposure.

### Assistance with report generation

Drafting imaging reports is another opportunity for AI to improve workflow efficiency (Fig. 1: report generation). Using structured templates and natural language generation, AI tools can automatically populate reports based on imaging findings and radiologist input^55^. These systems can flag inconsistencies, highlight missing details, and ensure adherence to reporting standards, helping radiologists produce complete and accurate reports more quickly^56^. The benefits of AI-assisted reporting are reflected in improved report quality^57^ and faster turnaround times^58^.

### Workflow systems integration

To maximize its impact, AI must integrate seamlessly into existing radiology workflow systems, such as picture archiving and communication systems (PACS), radiology information systems (RIS), and electronic health record systems (EHRs)^59^. Effective AI tools should function as intuitive extensions of these systems, reducing disruptions and ensuring interoperability. To determine success in such integration, we would look for reductions in system downtime, high user adoption rates, and low rates of interoperability errors. By aligning with existing processes, AI ensures that efficiency gains are achieved without adding unnecessary complexity to the workflow.

By optimizing existing workflow processes, AI offers a pathway to greater efficiency and productivity. These tools help radiologists work smarter and more efficiently, enabling them to handle increasing volumes of imaging studies while maintaining high standards of care. However, efficiency improvements alone may not be enough to address the challenges posed by rising demand and structurally limited supply. Meeting these challenges requires strategies to strengthen the radiology workforce and grow capacity.

## Capacity building

The transformative promise of AI lies in its potential to address supply challenges by expanding the ability to deliver high-quality services across the full scope of radiology, including diagnostic interpretation, patient communication, and education. Capacity-building initiatives can leverage AI to augment the capabilities and expertise of radiologists, improve workforce sustainability, and support the development of new skills and roles. While workflow optimization focuses on efficiency, capacity building aims to expand capabilities, reduce burnout, and ensure the workforce is resilient and prepared for future challenges.

### Burnout reduction and workforce retention

Excessive workloads in radiology contribute to burnout and job dissatisfaction. AI holds promise for reducing radiologist burnout by automating repetitive tasks, streamlining workflows, and enhancing diagnostic accuracy. AI tools can assist in image interpretation and generate preliminary reports, thereby allowing radiologists to focus more on complex cases and patient care (Fig. 1: radiologist support). However, the impact of AI on radiologist burnout is nuanced. In some cases, AI use has been associated with increased burnout, particularly among radiologists with heavy workloads or those with low acceptance of the technology^60^. To avoid unintended consequences, the implementation of AI should be approached with careful planning, including comprehensive training and thoughtful integration into clinical workflows to help ensure that AI enhances rather than disrupts the daily work of radiologists.

Teleradiology and remote work options can allow radiologists to balance their responsibilities more effectively, potentially reducing stress and improving job satisfaction^61^. AI can facilitate teleradiology and remote work by distributing workloads, assigning tasks, providing quality assurance, and improving coordination across disparate shifts and schedules. By monitoring work hours and case complexity to prevent fatigue, AI can help create a more fulfilling work environment and lessen cognitive burdens that lead to burnout.

### Training and education

AI can also play a positive role in workforce development by improving training and education for future radiologists (Fig. 1: education). AI may benefit trainees through greater personalization of case assignments, simulated training, and synthetic case generation. Natural Language Processing (NLP) tools can tailor worklist assignments based on trainee history to ensure balanced exposure across imaging modalities and subspecialties^62^. Automated case curation can support realistic training scenarios, such as on-call simulations, helping trainees build confidence while identifying areas for improvement^62^. Educational platforms powered by AI can provide trainees with in-depth information on imaging features, differential diagnoses, and research updates^62^. Additionally, generative adversarial networks (GANs) can generate realistic images of rare or underrepresented pathologies, supporting training in programs with limited case diversity^62^.

AI tools can also be used to monitor the progress of radiology trainees, helping educators to identify proficiency gaps and provide more focused teaching^63^. This targeted approach helps to ensure a more consistent, competency-based education across trainees. While these advancements cannot overcome the structural limitations on the number of new radiologists entering the field, they can enhance the effectiveness of training and help ensure the next generation of radiologists is highly capable.

### Patient communication

Patient communication is a critical, yet often overlooked, function of radiologists, and AI offers tools to transform this important aspect of care (Fig. 1: communication of results). AI can simplify complex imaging findings for patients by generating three-dimensional, context-rich visual explanations or using LLM chatbots to provide accessible, patient-friendly summaries^57^. These tools can also assist with administrative tasks such as scheduling and follow-up communication, freeing radiologists to focus on high-value interactions with patients. AI-based feedback can highlight empathetic responses and improve communication skills, fostering better doctor-patient encounters. By shifting radiologists’ time away from repetitive explanations and administrative duties, AI allows them to prioritize more impactful and rewarding aspects of their practices.

### Augmenting image interpretation

AI has rapidly evolved into a powerful tool for enhancing radiologists’ diagnostic capabilities (Fig. 1: imaging interpretation, reducing errors). A study to assess whether AI improves the detection accuracy of abnormalities on chest radiographs by radiologists with varying levels of expertise found that AI assistance led to an increase in sensitivity ranging from 6% to 26% (*P* < 0.001) for all readers, including thoracic radiologists, general radiologists, and radiology residents^64^.

When used as a second reader for mammography screening, AI matched or exceeded cancer detection rates compared to traditional double reading, suggesting that AI can reduce radiologist workload without compromising diagnostic performance^65–67^.

Advanced AI models are being developed to provide comprehensive interpretations of CT scans of the abdomen and pelvis^45^. These interpretations can be formatted as preliminary reports and integrated into radiology workflows to support greater efficiency or used as a second read for quality assurance.

A review of randomized controlled trials (RCTs) evaluating AI in clinical practice demonstrated that 81% of the trials reported favorable primary outcomes, particularly in diagnostic performance in image interpretation and clinical decision-making. However, many trials were single-center studies with limited demographic diversity, emphasizing the need for more diverse, multicenter trials to fully understand AI’s effectiveness and limitations in real-world healthcare settings^68^. Collectively, these findings highlight AI’s growing role as a valuable assistive tool in radiology, with the potential to improve diagnostic performance while enhancing efficiency.

Looking ahead, the next generation of AI models is expected to extend similar capabilities across a broad range of radiologic examinations, including MRI, X-ray, and ultrasound, with the potential to reduce radiologic errors. However, further research and rigorous clinical validation will be essential to ensure the safety, generalizability, and effectiveness of these approaches across diverse patient populations and practice environments.

## Conclusion

The integration of AI into radiology represents a transformative opportunity to address the workforce shortage through demand management, workflow efficiency, and capacity building. By curbing unnecessary imaging, AI can reduce health system strain and help ensure that imaging resources are used where they are most needed. At the same time, workflow efficiency improvements allow radiologists to handle increasing case volumes with fewer delays by streamlining processes, automating routine tasks, and optimizing resource allocation. Finally, capacity-building efforts leverage AI to augment radiologists’ abilities, support workforce development, and help ensure the sustainability of the profession.

These three approaches are interdependent. Reduction in overutilization and gains in workflow efficiency mutually reinforce to ease radiologists workloads. Better workflows, in turn, free radiologists for high-value tasks. Increasing radiologists’ capacity for high-value tasks amplifies efficiency gains, enabling them to more effectively meet growing demand. More productive, effective radiologists help to ensure long-term workforce sustainability. Together, these strategies demonstrate the potential of AI not just to fill gaps but to reshape radiology into a more effective and sustainable discipline.

## Data Availability

No datasets were generated or analysed during the current study.
